# Novel Approach in Rectovaginal Fistula Treatment: Combination of Modified Martius Flap and Autologous Micro-Fragmented Adipose Tissue

**DOI:** 10.3390/biomedicines11092509

**Published:** 2023-09-11

**Authors:** Ana Dimova, Ivana Erceg Ivkošić, Petar Brlek, Stefan Dimov, Tomislav Pavlović, Tomislav Bokun, Dragan Primorac

**Affiliations:** 1 St. Catherine Specialty Hospital, 10000 Zagreb, Croatia; 2 Faculty of Dental Medicine and Health, Josip Juraj Strossmayer University of Osijek, 31000 Osijek, Croatia; 3 School of Medicine, Josip Juraj Strossmayer University of Osijek, 31000 Osijek, Croatia; 4 Medical School, University of Split, 21000 Split, Croatia; 5 Department of Biochemistry & Molecular Biology, The Pennsylvania State University, State College, PA 16802, USA; 6 The Henry C. Lee College of Criminal Justice and Forensic Sciences, University of New Haven, West Haven, CT 06516, USA; 7 Medical School REGIOMED, 96450 Coburg, Germany; 8 Medical School, University of Rijeka, 51000 Rijeka, Croatia; 9 National Forensic Sciences University, Gandhinagar 382007, India

**Keywords:** Crohn’s disease, mesenchymal stem cell, micro-fragmented adipose tissue, modified Martius flap, rectovaginal fistula

## Abstract

In this paper, we introduce an innovative therapeutic approach for managing rectovaginal fistulas (RVF), by combining the modified Martius flap and micro-fragmented adipose tissue (MFAT) enriched with mesenchymal stem cells (MSC). This novel approach aims to deal with the difficulties associated with RVF, a medically complex condition with a lack of effective treatment options. We present the case of a 45-year-old female patient with a 15-year history of Crohn’s disease (CD). During the preceding eight years, she had encountered substantial difficulties resulting from a rectovaginal fistula (RVF) that was active and considerable in size (measuring 3.5 cm in length and 1 cm in width). Her condition was accompanied by tissue alterations at both the vaginal and rectal openings. Following her admission to our hospital, the patient’s case was discussed during both surgical and multidisciplinary hospital team (IRB) meetings. The team decided to combine a modified Martius flap with autologous MFAT containing MSCs. The results were remarkable, leading to comprehensive anatomical and clinical resolution of the RVF. Equally significant was the improvement in the patient’s overall quality of life and sexual satisfaction during the one-year follow-up period. The integration of the modified Martius flap with MFAT emerges as a highly promising approach for addressing CD-related RVFs that had historically been, and still are, difficult to treat, given their often refractory nature and low healing success rates.

## 1. Introduction

A rectovaginal fistula (RVF) is an abnormal, epithelium-lined communication between the vagina and rectum or only the anal canal. RVFs are less common than both simple and complex perianal fistulas that connect the rectum and/or the anal canal to the perianal skin [1]. In other words, fistulas of the rectum and anus most commonly connect those parts of the gastrointestinal tract and the skin of the perineum in lateral and dorsal projections. The fistulous tract passing through the rectovaginal (RV) septum and opening in the vagina (the definition of RVF) is not only less common, but also has lower healing rates with all available treatment options, both conservative and surgical. The presumed reason lies in the poorly vascularized rectovaginal septum. After obstetric or post-partum RVF, Crohn’s disease (CD) is the second most common cause of RVF. In the setting of CD, a chronic inflammatory process leads to ulcer formation, and finally forms a communication canal (fistula) with adjacent structures (internal organs or skin). CD-related fistulas have worse outcomes if there is vaginal involvement, and such fistulas more often demand proctectomy and/or fecal diversion [2,3,4]. CD-related RVFs have notoriously low treatment success rates, probably due to the combination of two reasons. Firstly, poor vascularization of thin tissue in the rectovaginal septum has significantly lower healing potential than tissues with an abundant capillary network. Secondly, pathophysiological mechanisms in CD lead to alterations in rectal and perineal tissue quality, with chronic inflammatory changes.

The conservative therapeutic options for treating CD-related RVF include antibiotics and various immunomodulators such as tacrolimus, azathioprine, 6-mercaptopurine, and biologic agents. However, these treatments need a long time to reach the desired healing outcome, have severe side effects, and can lead to relapse as soon as the treatment is ceased [5,6,7,8,9,10,11,12]. Infliximab is genetically engineered immunoglobulin 1 (IgG1), a monoclonal antibody that binds to TNFα (both to its soluble and transmembrane form) with high affinity. Given the fact that TNFα is a key proinflammatory cytokine in the CD pathophysiology cascade, infliximab is approved for the treatment of moderate to severe CD that has been refractory to conventional therapy such as systemic corticosteroids, 5-aminosalicylic acid (5-ASA), azathioprine and 6-mercaptopurine. Infliximab has been shown to be successful in treating fistulizing CD with different reported success rates. Infliximab has better response rates for simple perianal fistulas than for RVF, probably due to the above-mentioned characteristics of the rectovaginal septum and its poor vascularization [13,14,15,16]. Therefore, infliximab can be used to bridge inflammatory suppression before definite surgical management [17]. The most commonly applied surgical procedures are simple fistulotomy, long-term seton, and rectal flap closure [18,19]. The latter is contraindicated in cases of active proctitis or low compliance of the surrounding tissue [20]. Less common procedures are the gracilis muscle flap, Martius flap, rectal sleeve advancement flap, proctectomy, and diverting stoma [21,22,23,24]. Gynecologists have reported favorable outcomes with transvaginal repairs, but most of the reported cases had concomitant fecal diversion, eliminating the pressure gradient from the rectal side [25,26,27]. Injection of fibrin glue into the fistulous tract and bioprosthetic plug placement showed moderate success for simple perianal fistulas, and none for CD-related RVF [2,28,29,30,31,32].

Mesenchymal stem cells (MSCs) are a subset of adult stem cells found in various tissues, known for their robust regenerative capabilities and relevance to tissue regeneration research. Distinctive phenotypic and functional disparities set MSCs apart from other stem cells. The properties of MSCs include immunoregulatory, anti-inflammatory, anti-apoptotic, and anti-oxidative effects. Moreover, MSCs exhibit analgesic and angiogenic potential, heightening their therapeutic value. Within the sphere of medical innovation, our previous review article unveiled significant insights regarding an innovative approach grounded in micro-fragmented adipose tissue (MFAT) [33]. Executed through novel technology, this process selectively isolates adipose tissue, thereby preserving stromal-vascular fraction adipose-derived MSCs (AD-MSCs). The uniqueness of this methodology is underscored by the angiogenic, immunomodulatory, anti-inflammatory, anti-apoptotic, and anti-fibrotic properties of MSCs [34]. Even though mesenchymal stem cell (MSC) therapy has been studied for CD fistulas, most of those reports exclude RVF or have sporadic cases of RVF and offer no combined surgical procedure and MSC application. To the best of our knowledge and according to the up-to-date literature, this is the very first time that the modified Martius flap was combined with the MFAT application for RVF treatment.

## 2. Case Presentation

We present a case of a 45-year-old female Caucasian patient with CD-related low RVF. To the best of her knowledge, her son, who was diagnosed with CD at the age of 16 years, is the only family member with the illness. She had no other lifestyle risk factors, she is non-smoking, lives in a moderately polluted city on the Adriatic coast, and is used to the Mediterranean diet. She does not consume alcoholic beverages. She had two uncomplicated pregnancies, both before her diagnosis, both delivered vaginally with lateral episiotomy. The recovery after both deliveries was uneventful. Her other medical history was unremarkable. She had been diagnosed with CD at the age of 30. The patient’s CD was classified according to the Montreal classification as A2, L2, B1 (age at the diagnosis 17–40 years, colon location, non-stricturing and non-penetrating behavior). The initial treatment strategy consisted of oral antibiotics (ciprofloxacin 2 × 250 mg for one month, and metronidazole 3 × 400 mg for 3 months), combined with oral mesalazine at an initial dosage of 4.5 mg daily, and a maintenance dosage of 1.5 mg daily. A rectal route of mesalazine administration was tried and ceased after 2 months, due to the local irritation that caused the patient’s poor compliance. In the third trimester of the year 2015, and for the first 6 months of the year 2016, azathioprine was used at a dosage of 2 mg/kg of body weight, with no success. During that period, she had higher fecal calprotectin levels and uncontrolled diarrhea, with continuous weight loss. From the 2015 onwards, she also used methylprednisolone with a constant dosage increase of up to 48 mg per day. Seven years after the onset of CD, she developed a perianal abscess which drained spontaneously into the vagina, leaving a wide RVF behind. Initial conservative treatment was changed to infliximab, and CD re-classified to A2, L1, B3p (age at the diagnosis 17–40 years, colon location, penetrating behavior, perianal disease). Infliximab was administered intravenously (5 mg/kg) at 0, 2, and 6 weeks as an induction regimen, which resulted in almost complete remission of CD and a quiescence clinical period, apart from the persistent wide RVF. Infliximab was continued at a maintenance dosage of 5 mg/kg IV given every 8 weeks. Even though other CD symptoms ameliorated, our patient had daily stool and flatus passage per vagina continuously over a period of 8 years, which deteriorated her psychosocial and sexual esteem and seriously affected her quality of life and sexual functioning. One year before our procedure, she underwent one failed attempt at tract curettage and seton placement. The patient was motivated to undergo other possible repair attempts, but was not satisfied with persistent fistula and long-term seton.

### 2.1. Clinical Findings

Our patient had a 3.5 cm-long and 1 cm-wide RVF with callus tissue changes in both the vaginal and rectal openings. Even though the rectal mucosa showed no active proctitis, the fistulous tract was thick, with barely any intact tissue in the rectovaginal septum and very poor compliance of the peri-fistulous mucosa.

### 2.2. Diagnostic Assessment

**Figure 1:**
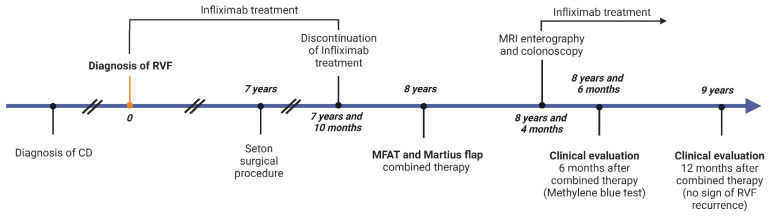
Timeline.

Our patient had a wide RVF, easily seen on inspection in ambulatory settings. Apart from the described RVF, preprocedural MRI enterography showed no other active fistulas, and CD remission was marked on colonoscopy findings as well. MRI and colonoscopy, as well as the methylene blue test, were performed 4 and 6 months postoperatively, respectively (Figure 1).

### 2.3. Preparation of MFAT Containing MSC

The surgical aspect of the procedure took place within an operating theater setting. To ensure sterility, the abdominal skin underwent a series of treatments. Firstly, it was treated with an antiseptic solution known as Dermoguard^®^ (Antiseptica, Pulheim, Germany). Following this, the area was rinsed using Aqua Pro injection solution (HZTM, Zagreb, Croatia) and meticulously dried. Subsequently, the skin was disinfected utilizing Skin-Des^®^ solution (Antiseptica, Pulheim, Germany). A solution consisting of 250 mL of the saline solution prepared with 40 mL of a 2% lidocaine solution (Lidokain^®^, Belupo, Koprivnica, Croatia) and 1 mL of epinephrin hydrochloride (1 mg/mL) (Suprarenin^®^, Sanofi-Aventis, Berlin, Germany) was injected into the abdominal subcutaneous adipose tissue. Subsequently, the aspiration step was undertaken, employing a standard lipoaspiration technique, as described in our previous paper [35]. Lipoaspiration was performed through a bilateral symmetrical skin incision on the abdominal wall only a few millimeters in width, following the injection of Klein’s solution in the subcutaneous fat plane. The lipoaspirate was then processed mechanically in a closed, low-pressure cylindrical system to obtain MFAT containing MSC (Lipogems; Lipogems International SpA, Milan, Italy). From 90 mL of aspirated fat, we gained 10 mL of purified MFAT.

### 2.4. Therapeutic Intervention

**Figure 2:**
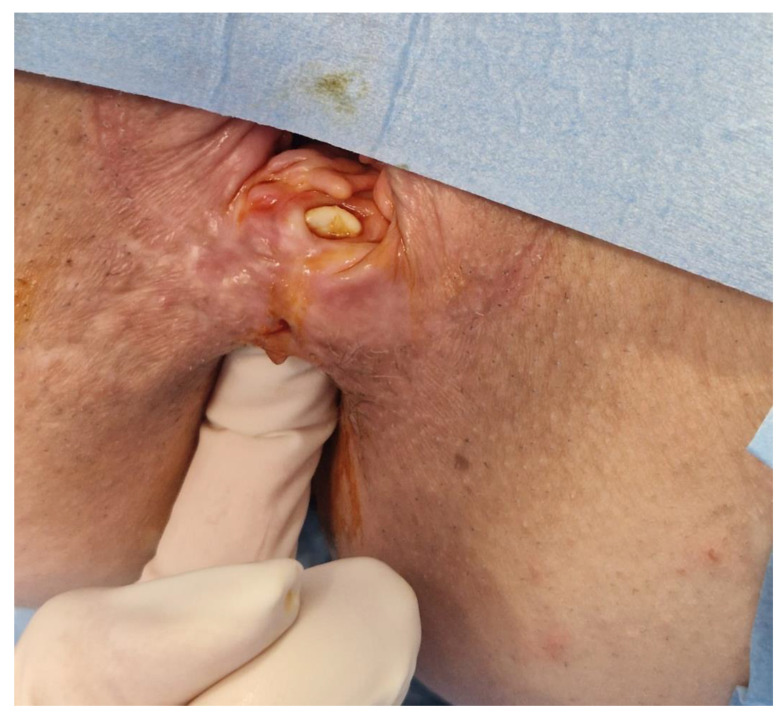
The figure shows the start point of our procedure, with a large RVF completely consuming the RV septum and its callus vaginal opening.

We combined the modified Martius flap taken from labiummajor and autologous MFAT containing MSC. The Martius flap was first described in 1928 for urethrovaginal reconstruction purposes, using bulbocavernosus muscle. The modified procedure is based on the use of a vascularized labium major adipose tissue flap without muscle mobilization. Transposition of well-perfused tissue onto the area of the previously damaged rectovaginal septum and RVF can provide neovascularization and the formation of granulation tissue in the rectovaginal septum. Pitel et al. [36] reported a series of twenty patients treated with the Martius flap procedure for RVF, with CD being a predominant cause. The reported overall success rate reached 50% in CD patients, with low morbidity and no negative effect on the quality-of-life score. Infliximab therapy was discontinued eight weeks before this procedure because of its possible mitigation of the immunomodulatory effects of MSCs [37,38,39]. The procedure was performed under general anesthesia. The patient was placed in a lithotomy position. We identified a 1 cm-wide and 3.5 cm-long low RVF, with a very thin and short rectovaginal septum above and below the fistulous tract (Figure 2). The rectal opening was above the dentate line, and the vaginal one was in the introitus, with a thick RVF tract and opening edges, and low surrounding tissue compliance.

**Figure 3:**
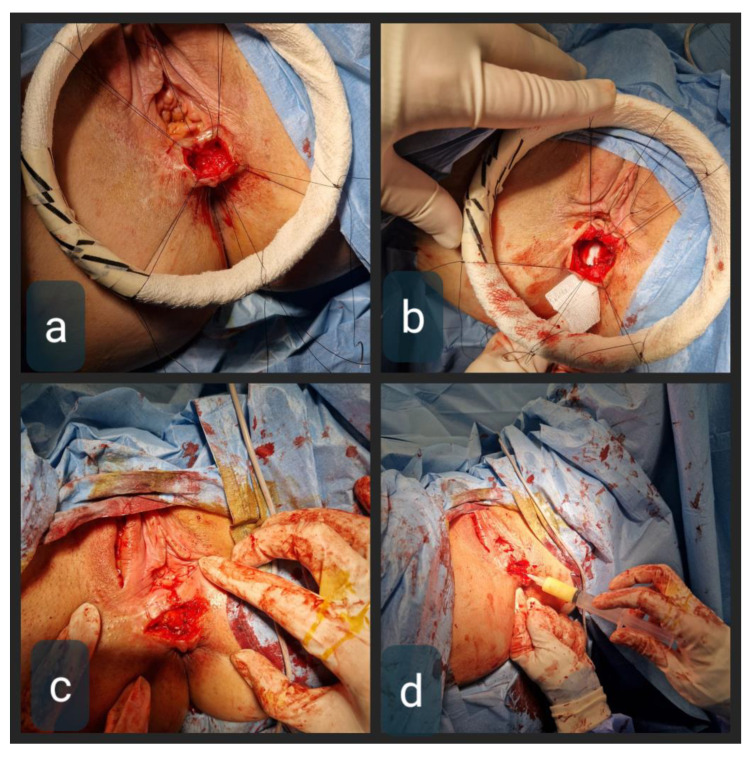
(**a**) RVF canal isolated from the surrounding tissue; (**b**) rectal opening after fistula removal, gauze in the rectum showing a large rectal mucosal defect; (**c**) Martius flap sealed in the rectovaginal septum; (**d**) MFAT application around the flap and both rectal and vaginal opening.

**Figure 4:**
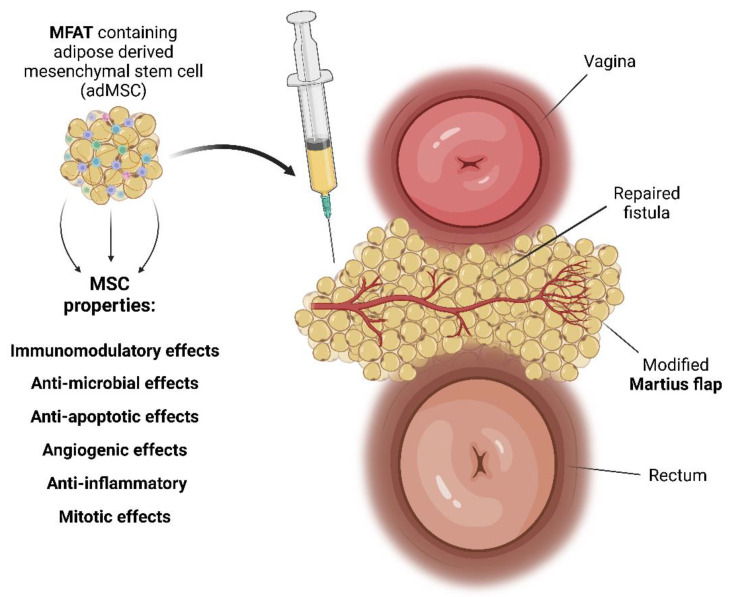
The figure illustrates the surgical approach (modified Martius flap) and injection of micro-fragmented adipose tissue (MFAT) containing mesenchymal stem cells (MSCs) for the treatment of rectovaginal fistula. Using tissue from the patient’s labia majora, the modified Martius flap aids in healing. To speed up the healing process, stem cells with immunomodulatory, anti-microbial, anti-apoptotic, angiogenic, anti-inflammatory, and mitotic properties are injected into the flap [35,40,41,42]. In our patient, this combination proved to be an effective treatment and improved her quality of life.

Through the horizontal perineal incision, the rectovaginal septum was opened and RVF was mobilized below and high above the tract, to provide for not only clear fistulectomy but also a tension-free multi-layered closure. The tract itself was then excised, with the rectal opening sutured by 3–0 Vicryl interrupted sutures. The vaginal opening was narrowed by sutures in the same manner but left open for 3 mm to allow for drainage. The vertical incision in the right labium major was made from the level of the mons pubis to the bottom of the labium. The subcutaneous fat flap was mobilized by ligating its superior vessels, whilst the lower blood supply of the flap was preserved. The next step was tunneling the subcutaneous space from the labium major flap donor site to the rectovaginal septum. Its adequate width was necessary to ensure tension-free tissue interposition with good rotation and uncompromised blood flow, avoiding any pressure and kinking. The flap was sealed in place with interrupted 4–0 Vicryl sutures (Figure 3). The final step was the application of MFAT containing the MSC around the flap in the rectovaginal septum (Figure 3 and Figure 4).

Given the fact that the area offers low volume compliance, in order to avoid excess pressure to the flap blood supply, we applied only 6 mL of MFAT through a 16 Gauge needle, in four consecutive slow applications over the course of 10 min. The labial incision was closed in layers with absorbable interrupted sutures and drained for 24 h. The perineal incision was not drained. The patient was discharged 36 h postoperatively with no antibiotic therapy.

### 2.5. Follow-Up and Outcome

**Figure 5:**
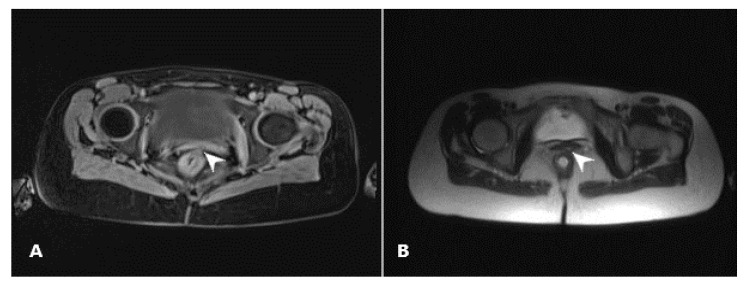
(**A**) MRI of the pelvis—axial fat-suppressed T1-weighted image with an arrowhead pointing to an abnormal fistulous connection between the rectum and the vagina; (**B**) MRI of the pelvis—axial T2 weighted image after treatment with an arrowhead pointing to no abnormal fistulous connection between the rectum and the vagina.

**Figure 6:**
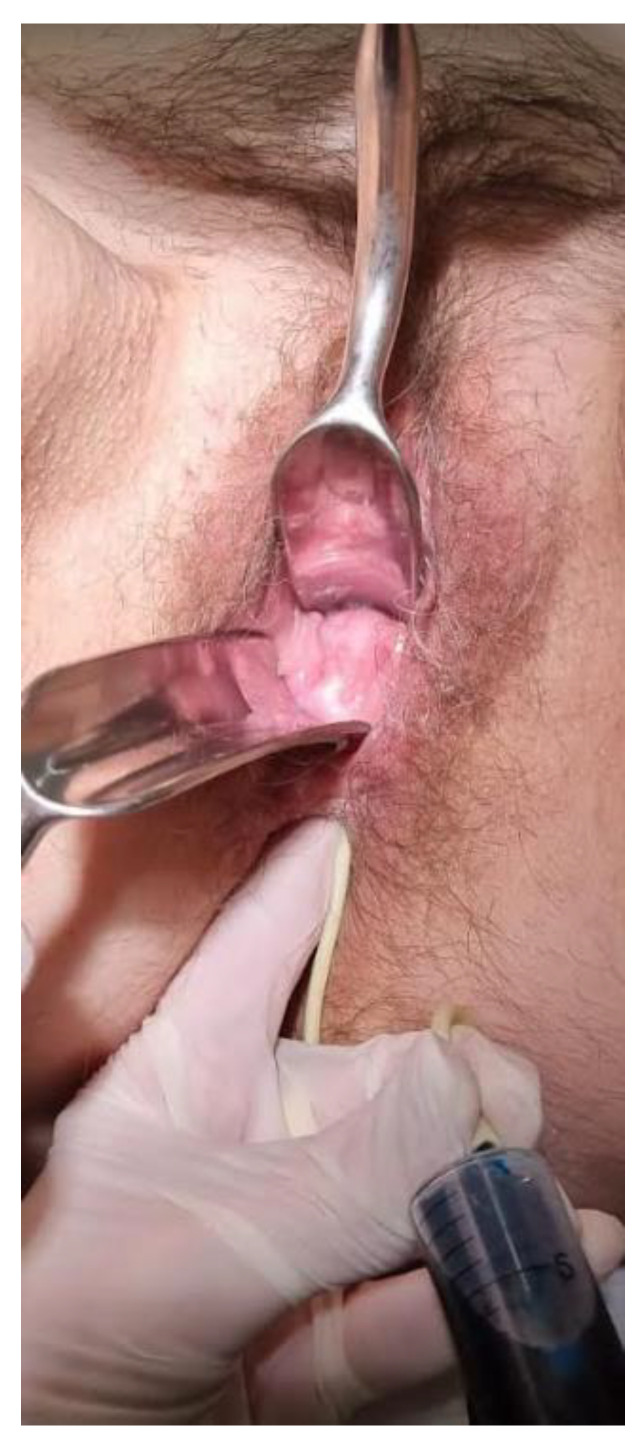
Using a Foley catheter (26 French), a controlled application of fifty milliliters of methylene blue was directed into the rectum. Concurrently, the vaginal area was observed through a speculum, with the entire process recorded on video (Supplementary Video S1). It is important to note that there was no observable presence of dye within the vaginal region. The Sexual Satisfaction Index (SSI) and Short Inflammatory Bowel Disease Questionnaire (SIBDQ) were measured before and 4 months after the procedure. There was a significant increase in both SSI (14 compared to 20) and SIBDQ (36 compared to 61).

Apart from severe nausea on metronidazole, which stopped immediately after the discontinuation of the drug, the patient had an uneventful recovery. Serosanguinous discharge from the vaginal opening was expected and ceased after 3 weeks. Her analog pain scale score after this procedure was 2/10 on the first postoperative day, 1/10 one week postoperatively, and 1/10 one month postoperatively. Complete healing was confirmed via 3 objective methods: 4 months after the procedure with MRI enterography (Figure 5) and colonoscopy, and 6 months after with methylene blue installation. Fifty milliliters of methylene blue were applied under pressure in the rectum using a Foley catheter (26 French) while monitoring the vagina per specula, captured on video. No dye was found in the vagina (Figure 6, Supplementary Video S1). Objective methods were compliant to complete clinical quiescence. The vaginal opening site is at this point, six months later, noticeable as mucosal denivelation, but completely closed—confirmed by all of the above-mentioned methods.

No flare-up of CD was marked during the infliximab cessation, controlled by monthly outpatient visits with clinical examination, fecal calprotectin, SE, leukocyte, and CRP levels. Three months postoperatively, infliximab was commenced again, following the colonoscopy.

To this date, 12 months after the procedure, the patient has no signs or symptoms of RVF recurrence. She has completely restored her sexual life and her self-esteem.

## 3. Discussion

Even though the size, localization, and etiology of RVFs are obvious characteristics that determine diagnostic and therapeutic actions, and are included in most classification attempts, there is no generally accepted classification of RVFs. From a surgical perspective, probably the most important distinction is one between high and low rectovaginal fistulas. Low fistulas open at or just above the dentate line, and the vaginal opening is usually found on the lower part of the vaginal tube. As such, they can be accessed through the vagina, anus, and perineum. Concerning size, fistulas can be classified as “small” if they are less than 0.5 cm in length, “medium” from 0.5 to 2.5 cm, and “large” if they are longer than 2.5 cm. Fistulas are considered “complex” if they are large, highly situated, previously unsuccessfully surgically treated, or are caused by an underlying inflammatory pelvic process (for example IBD or diverticulitis) or irradiation. Our patient had a complex and large fistula. Similar to published reports, long-term seton resulted in patients’ poor satisfaction and compliance [43].

The Martius flap was first described almost a century ago for urethrovaginal reconstruction [44]. The procedure was later modified as a vascularized labium major adipose tissue flap and reported for use in RVF treatment [45,46]. Its rationale is in the transposition of well-perfused tissue onto the area of previously damaged rectovaginal septum, where it can occupy the “dead space”, and provide neovascularization and the formation of granulation tissue. The reported overall success rate reached 50% in CD patients, with low morbidity and no negative effect on the Quality-of-life score.

Mesenchymal stem cells (MSCs) are multipotent adult stem cells that have the ability to differentiate into a variety of cell types (adipocytes, osteoblasts, myocytes, chondrocytes, etc.) dictated by the given milieu [35,40,41,42,47,48,49]. Regarding origin, MSCs can be extracted from the bone marrow (BM-MSCs), umbilical cord MSCs (UC-MSCs), amniotic fluid SCs (AF-SC), placental MSCs (P-MSC), menstrual blood SCs (Men-SC), breast milk, cervix, dental tissue, synovial tissue, and fluid, or can be adipose-derived MSCs (AD-MSCs) and amnion-derived MSCs. MSCs can successfully migrate into injured tissues and inflamed areas, where the multipotent differentiation ability is beneficial. Furthermore, they have the ability to create daughter cells that share parent cell characteristics, which is necessary to remain in the “MSC pool” in the body. Their strong immunomodulatory role is achieved by suppressing both the proliferation and activation of immune cells, as well as cytokine and growth factor secretion. MSCs have shown paracrine properties regarding immunoregulatory, anti-inflammatory, anti-apoptotic, analgesic, angiogenic, and anti-oxidative effects [40,41,47]. Via polychromatic flow cytometric analysis, earlier, we determined the phenotypes of the CD45 subpopulation in autologous micro-fragmented samples containing the stromal vascular fraction (SVF). The most predominant cells include endothelial progenitors (EP) (CD31+ CD34+ CD73± CD90± CD105± CD146±), mature endothelial cells (CD31+ CD34− CD73± CD90± CD105− CD146±), pericytes (CD31− CD34− CD73± CD90+ CD105− CD146+), transitional pericytes (CD31− CD34+ CD73± CD90+ CD105− CD146+), and supra-adventitial-adipose stromal cells (CD31− CD34+ CD73high CD90+ CD105− CD146−) [48,49]. On the other hand, MSCs express very low levels of surface antigens and therefore do not trigger an immune response, allowing their usage as therapeutic agents in regenerative medicine [50,51,52,53]. Research indicates that MSCs exhibit anti-inflammatory properties that have the potential to regulate pain by reducing inflammation, modulating astrocyte reactivity, and the microglia phenotype, and are even applicable for the treatment of neuropathic pain [53]. The antimicrobial activity of MSCs is achieved through the upregulation of LL-37, which is amplified by bacterial stimuli and has been demonstrated to decrease bacterial growth [54]. Given their anti-inflammatory and immunomodulatory properties, several clinical studies have been conducted to evaluate the safety and efficacy of MSC therapy for Crohn’s disease. While the results have been mixed, reporting on different MSC origins at routes of application, some studies have shown promising outcomes, and most of them confirmed the safety of MSC application. For example, a phase 2 clinical trial published in 2015 found that treatment with MSCs was associated with significant improvement in Crohn’s disease activity scores and endoscopic scores compared to placebo. This randomized, double-blind, placebo-controlled trial evaluated the safety and efficacy of allogeneic bone marrow-derived mesenchymal stem cells (bmMSCs) in the treatment of Crohn’s disease [55]. Garcia-Olmo et al. indicated that, in cases of perianal CD, the local administration of MSC is superior to the systemic administration [36]. MFAT with MSC has shown encouraging results in an intralesional application for perianal CD fistulas by Laureaty et al., but with RVFs excluded [56]. Further research is needed to establish the optimal dose, route of administration, and long-term efficacy and safety of MSC therapy for Crohn’s disease [55,56,57].

Given the fact that a couple of the mentioned algorithms for the treatment of RVF suggest fecal diversion in surgical re-do attempts, and our patient had no diverting stoma formation, we suggest the further evaluation of local MFAT-MSC application in cases of repeated surgeries in order to lower stoma formation rates, as well as to lower postoperative inflammation and pain and improve chances of any RVF surgical procedure [4,8,58].

We noticed almost no postoperative edema, and almost no pain (Visual analog scale 1-2/10), at the both fistula and flap donor site, and even though no antibiotics were administered, and no postoperative erythema, induration, or any signs of inflammation occurred, which we believe to be closely related to an anti-inflammatory effect of MSCs and LL-37 [54]. Since the surgical part of the procedure left the vaginal opening of the RVF partially open, we suggest that MSCs played a role in its closure. A possible flare-up of CD after the infliximab cessation prior to the MSC application has been reported [33]. Our patient tolerated 6 months of infliximab cessation well, with no clinically relevant worsening of CD. Clinical trials in the future may investigate the possible continuation of infliximab in the entire periprocedural period in the application of MFAT/MSC. We find visual analog scale scores to be particularly interesting, given the fact that an extensive procedure resulted in less pain than simple curettage and seton placement (2/10 vs. 4/10, respectively), which may be due to the effect of MSCs [53].

In conclusion, all studies available in up-to-date literature analyzed MSC application for complex perianal CD-related fistulas, after previously failed medical and surgical healing attempts. RVFs are predominantly excluded from such trials, and/or MSC application is presented as a sole salvage approach. We suggest that MSC therapy should be considered earlier in the patient approach timeline, as well as combining a surgical attempt with local MSC application, rather than dividing these treatment options. We believe that MFAT-MSC application raises the overall success rate, with good patient compliance, possibly less postoperative pain, minimal additional effort, and no significant adverse effects.

This is a promising but single case report, and further investigation is needed to adequately analyze the proposed novel approach.

## 4. Patient Perspective in an Original Statement

After the surgery combined with stem cell therapy, I was surprised how the level of the pain was low, especially compared to previous surgeries and seton placement. I had only mild pain in my right big labium. Apart from nausea on the antibiotic, I had no complaints, and it stopped immediately after my doctor approved the antibiotic withdrawal. I was very discouraged by different specialist consultations over the past 8 years who repeatedly told me that I would have to live with this fistula for the rest of my life. Maybe it was a long shot, but I am extremely glad I decided to go through this procedure now. Better ever than never. However, striking it may be, I had no sexual intercourse over many years, due to the unpleasant discharge, resulting in shame, and generally low sex mood as a result of it. My quality of life in general was very low. This is an overwhelming turnover for both my personal, and satisfaction in my married life. My self-esteem is revived and I feel like a new woman. Big thanks to the medical team of St. Catherine’s Hospital and especially to my one and only dr. Dimova.

## Data Availability

The data presented in this study are available on request from the corresponding author.

## Supplementary Materials

The following supporting information can be downloaded at: https://www.mdpi.com/article/10.3390/biomedicines11092509/s1.
